# Improving the Environmental Footprint through Employees: A Case of Female Leaders from the Perspective of CSR

**DOI:** 10.3390/ijerph182413082

**Published:** 2021-12-11

**Authors:** Shilong Wei, Muhammad Safdar Sial, Wenxia Zhou, Alina Badulescu, Daniel Badulescu

**Affiliations:** 1 School of Labor and Human Resources, Renmin University of China, Beijing 100872, China; shilongwei@ruc.edu.cn; 2 Department of Management Sciences, COMSATS University Islamabad (CUI), Islamabad 44000, Pakistan; safdarsial@comsats.edu.pk; 3 Department of Economics and Business, Faculty of Economic Sciences, University of Oradea, 410087 Oradea, Romania; abadulescu@uoradea.ro (A.B.); dbadulescu@uoradea.ro (D.B.)

**Keywords:** CSR, pro-environmental behavior, healthcare sector, ethical leadership, gender

## Abstract

Environmental quality strongly depends on human behavior patterns. Many environmental challenges are rooted in human actions, and thus, it is believed that these problems can be reduced through the promotion of pro-environmental behaviors (PB). Owing to this reality, the current study aims to reduce the environmental footprint of a hospital by promoting its employees’ environment-specific behavior via corporate social responsibility (CSR) and ethical leadership (EL). More importantly, the study also considered the role of female leaders in the proposed relationship. The current study collected the data from the respondents employed in different hospitals of a developing economy through a questionnaire (paper-pencil method). A total of 489 valid responses were collected, which were analyzed by employing the structural equation modeling (SEM) technique. As per the current study’s findings, there is a positive relationship between CSR, while EL mediates between CSR and PB. Likewise, the moderating role of female leaders in the proposed relationship was more significant than that of male leaders. More specifically, the study’s findings have considerable theoretical and practical implications, as it opens paths for researchers to further investigate the applicability of different dimensions of CSR and the role of gender in environmental sustainability. It provides insight to policymakers on how to restructure their CSR preferences, priorities on the environment, and gender differences.

## 1. Introduction

Many organizations worldwide cause contamination of the environment, which has become a concern for researchers and policymakers [1]. Environmental dilapidation adversely affects living beings and even nonliving beings around. Specifically, in some countries such as Saudi Arabia (KSA), greenhouse gas emissions have increased by 225% since 1990. More specifically, during recent years, KSA produced more than 490 million metric tons of air pollution and has been blamed for contributing 1.47% of greenhouse gases globally each year [2]. These Hypothesized framework s paint a bleak picture of the country from the perspective of a sustainable future and call for extraordinary measures at each level. Currently, many developing countries vulnerable to changing climatic conditions, as most of its ecosystems are sensitive.

Moreover, the nation has been facing increasing temperatures, floods, and droughts. The country has faced huge natural disasters stemming from environmental disorders [3]. Hence, there is an urgent need to take emergency measures in order to mitigate this vulnerability.

Industries, including the healthcare sector, have a substantial impact on the environment as they contribute to environmental issues such as climate change [4], waste [5], deforestation [6], and water and air pollution [7]. In this regard, hospitals particularly emit hazardous pollutants of various classes, potentially causing several diseases, including, among others, lung cancer. The release of chemical and other types of pollutants through hospital waste to the environment without scientifically managing it creates critical environmental issues and potentially causes dangerous diseases. In return, even hospitals themselves become vulnerable to adversities of this kind of environment, which negatively affects hospital performance by endangering patients’ lives and affecting treatment processes.

In order to mitigate environmental deterioration, organizations need to work to reduce their environmental footprint. Consequently, researchers around the world started introducing concepts, such as green human resource management [8], green organizational performance [9], green marketing [10], and green change [11]. Since tendencies and definitions of organizational success are changing, it is not enough to earn profits for survival and growth, but it becomes imperative for modern organizations to contribute to the environment positively [12].

As industries are creating life-threatening pollution on earth, they must play a vital role in controlling it. This is why corporations in the current era are under immense pressure from different stakeholders (customers, employees, the public, or even the shareholders) to improve their environmental footprint and stewardship [13]. Whereas efforts to reduce environmental dilapidation at government levels occur in several states, the striking observation of different scholars is that such efforts will be unsuccessful without improving the individual behavior towards the environment [14,15,16]. Thus, promoting the pro-environmental behavior among employees of a corporation is of utmost importance for a corporation to improve its overall environmental footprint. Therefore, one of the primary objectives of the current study is to explore the factors that can improve the pro-environmental behavior of employees.

Wang et al. [17] revealed an immense increase in the levels of greenhouse gases and CO_2_ emissions in recent years. Concerns about economic growth, environment, and social interaction are not new, but the pressure on organizations to act in a socially responsible manner is increasing faster than ever before [18]. Meanwhile, the importance of corporate social responsibility (CSR) has increased the need to conduct activities differently by integrating the community’s issues and activities in a targeted, socially responsible, and economical way [19]. Researchers have shown a severe concern for CSR, which is considered one of the most important phenomena for modern enterprises from the perspective of environment management [20,21]. Firms have the right to sell goods or services to consumers, but they are also responsible for conducting their activities in a socially responsible manner.

Corporate social responsibility generally includes philanthropy, environment conservation, diversity and labor practices, and volunteerism. However, in the context of the healthcare sector, the potential role of CSR to mitigate the environmental footprint of a hospital is not well-explored. In a healthcare context, CSR activities were directed on charity and welfare-related activities such as free treatment, including free or subsidized surgeries, lab tests and investigations, surgical equipment, medicines, and even food and accommodation to patients [22,23]. Though there have been a few recent studies from the healthcare sector [21,24] to propose CSR as an enabler to improve the environmental footprint of a hospital, such studies are sparse, implying that more research is required to explore the importance of CSR from the perspective of environmental management. Therefore, another objective of the current study is to investigate the relationship between CSR and employees’ pro-environmental behavior.

Recently, scholars have acknowledged the importance of ethical leadership to shape employees’ behavior. In particular, it has been reported that an ethical leader is likely to influence the discretionary behavior (not formally required for a job) of an employee [25,26]. Specifically, there exists a positive relationship between ethical leadership and employees’ pro-environmental behavior. More specifically, in some recent studies, the mediating role of a leader to spur employees’ discretionary behavior has been reported [27]. In the current context, the pro-environmental behavior of an employee is also a discretionary behavior; therefore, it will be interesting to investigate the mediating role of an ethical leader between the relationship of CSR and employees’ pro-environmental behavior. Thus, investigating this mediation effect is another important objective of the current research.

According to the social role theory [28], several scholars have concluded that gender differences in attitudes and behaviors will continue to emerge in different settings and cultures. Common sense suggests that women focus more on the role of a guardian than men and are interested in learning and nurturing social behaviors, such as helping and caring for their colleagues [29,30]. In the current context, the role of female leaders to transfuse pro-environmental behavior among employees has a high significance. Hence, the last objective of the current study is to explore the moderating role of a female leader in the above-proposed relationships.

The proposed framework of the current study was applied in the healthcare sector. This sector was considered relevant to serve the purpose of the current survey due to the following specific reasons. First, as stated earlier, hospitals are a significant pollutant. In the case of developing countries, hospitals are blamed for producing a large amount of solid waste, including plastic, glass, metal, food, and others [31]. Unfortunately, this waste ends up in landfills without any recycling. Therefore, the promotion of environment-specific behavior among hospital employees may be helpful in reducing this level of environmental dilapidation on the part of hospitals.

Yet, another reason for choosing the healthcare sector was the considerable presence of females. Given that females almost constitute half of the country’s population [32], their contribution to employment is just above 30% [33]. Moreover, healthcare and teaching are the only two sectors with a significant female contribution in the country. Thus, the consideration of female leadership to spur pro-environmental behavior among employees in the healthcare sector will be of prime importance from the perspective of environmental management for emerging economy.

The present research is an action with three definite contributions to the available literature. Firstly, the current study attempts to project CSR beyond the charity domain to improve an organization’s environmental footprint. In this regard, a majority of previous studies on CSR were conducted from a philanthropic orientation, including providing some temporary relief to deprived individuals [34,35]. The current study attempts to create an understating that CSR is a mechanism that can be employed for specific sustainable outcomes, including environmental well-being.

Secondly, CSR is viewed as a macro or institutional level phenomenon, and less attention is paid to individual involvement in CSR. In this study, it is believed that behaving in a socially responsible manner at an employee or individual level can yield equal or more achievements than at the macro level. This is more relevant to the population because more environmental issues arise due to individual-level carelessness. Thirdly, unlike other industries, hospitals are organizations with significant female participation. We highlight the existence of a gender role difference, and that the above-mentioned gender differences influence the realization of environmental objectives. Thus, the focus of the present research is to explain whether environmental issues can be managed using CSR activities.

The remainder of the current study is composed of four different sections. For example, the next section deals with related theories and literature to frame different hypotheses. It is followed by the methodology section, in which the authors have discussed the issues related to sample and data collection procedures, followed by the result section, which deals with the analysis of the data by employing different statistical tests. The last section belongs to the discussion, implications, and conclusion of the current survey.

## 2. Literature Review

### 2.1. Theoretical Framework

Social exchange theory [36] and social role theory [28] have been employed to provide theoretical grounds for this study. Social role theory suggests that men and women tend to do different roles aligned with social structures and are supposed to be judged according to a set of different standards for how to act. Social role theory explains that recurring roles and activities are divided into different socially determined categories wherein each individual is expected to perform a predetermined role. Likewise, the study argues that both males and females have different roles and perform different in a social setting.

Social exchange theory (ST) argues that social behaviors are outcomes of specific exchange processes. Specifically, the objective of such an exchange process is to maximize benefits. Various scholars have primarily used this theory in positive human psychology and individual behavior research [37,38,39]. Accordingly, ST suggests that individuals are likely to exchange benefits received from others with benefits. This exchange process is likely to continue until the benefits from one party outweigh the costs. In an organizational context, both CSR and ethical leadership promote ethicality in a workplace, and therefore, the employees assume such ethical practices as a benefit to society and the workforce. Thus, the employees also respond positively by exchanging ethical practices of a socially responsible organization with benefits. All this process motivated them to respond with a positive behavior in the best interest of the organization.

### 2.2. CSR and Pro-Environmental Behavior

The phenomenon of CSR is receiving mounting importance in the business world and is generally having a great effect on social life. It was also argued in the available literature that social and cultural factors can have a significant impact on the CSR activities of an enterprise [40,41]. CSR in developing countries has become an important business imperative for the social survival of enterprises [34,42]. The manifestation of socially responsible business practices for different stakeholders (employees, customers, and even shareholders, among others) has now become a priority agenda. Given that KSA is an oil-dominated economy with strong state power and ruling dynasty, the country has different sustainability and social responsibility perspectives [43]. Accordingly, Saeidi [44] mentioned that local endogenous CSR features in KSA have their origin in the country’s cultural, social, and religious (Islamic) environment. This implies that KSA’s social, cultural, and Islamic values are detrimental to CSR activities in the country. Responding to this, Murphy et al. [45] carried out a survey to explore whether CSR perception in KSA differs from other Muslim states. They showed that KSA’s rentier-state welfare accounts for the differences compared to other states. Additionally, they also revealed that females were stronger advocates for CSR implementation. In this regard, religion and contextual factors can also lead to differences in CSR perception. Realizing this, Koleva [46] conducted a study in the region of the Middle East and found that there was a significant impact of religious context in shaping the CSR perceptions of individuals. Moreover, the study also revealed that CSR perceptions were grounded in social and altruistic actions that align with the local cultural traditions and Sharia laws.

Given the current vulnerable climatic conditions throughout the globe, the corporations in the current age are expected to create a minimum negative impact on the environment.

At the same time, organizations are expected to have a caring attitude towards the environment by implementing environment-friendly processes and aligning their workforce with activities related to environmental performance [47]. Organizations embark on various approaches, including CSR activities, to tackle environmental challenges. Organizations have many compulsions to engage in CSR-related activities, such as competition [48] and the expectations of different stakeholders [49]. Organizations can enhance their environmental performance by adding to CSR activities various environment-related goals, such as low energy consumption and waste management systems [50]. Different stakeholders, including suppliers, customers, owners, competitors, employees, and government agencies, compel organizations to direct their CSR activities towards managing environment-related issues [51].

Researchers believe that CSR is a potential management strategy that can yield outstanding outcomes for organizations to respond to nature [52]. CSR is a context-specific and complex concept that is viewed differently in different societies of the world, and this could be why there is no unanimous and universally accepted definition of CSR. In this regard, the current study agrees with the frequently cited definition of CSR by Carroll [53], who says that CSR includes four sets of responsibilities for a business: economic, legal, ethical, and philanthropic commitments.

Existing literature in the vicinity of CSR is mainly linked with the effect of CSR programs on the financial performance of an organization [54,55]. Recently, researchers have investigated CSR’s role in environment management [56,57]. Studies in other contexts also confirm the positive association of CSR with environment management [58,59]. Given that enterprises are significantly responsible for the current climatic conditions throughout the globe, it is quite possible to improve an organization’s environmental footprint by promoting pro-environmental behavior among employees. Such environment-specific behavior has a clear manifestation for an enterprise, as indicated by several scholars [60,61]. More specifically, in healthcare settings, in different recent studies, it has been reported that there is a positive relationship between CSR and pro-environmental behavior. For example, the study of Molnár et al. [21] stated that the CSR orientation of a hospital enterprise has a positive relationship with employees’ pro-environmental behavior. Similarly, the same positive relationship was reported in a recent study by Ahmad et al. [24]. Moreover, the study of Nichols et al. [62] also acknowledged the same in the healthcare sector UK.

The authors believe that CSR at the level of employees could be even more effective in managing environmental issues. Healthcare organizations, particularly hospitals, are labor-intensive organizations and can use their employees to manage environmental issues. Emphasizing CSR from the employees’ perspective would yield a more productive outcome because each individual will be responsible for their environment-related roles. Thus, there exists a positive relationship between CSR and employees’ pro-environmental behavior. Therefore, the following hypothesis is formulated:

**Hypothesis** **1** **(H1).** *There exists a positive relationship between CSR and employees’ pro-environmental behavior*.

### 2.3. CSR and Ethical Leadership

Following Doh and Stumpf [63], studies in leadership, business ethics, and CSR developed independently. More recently, the business model is beginning to recognize that leadership can be an important factor in developing a business environment [64]. How to improve the leader–follower relationship through ethical leadership emerged as a research subject. The foundations of ‘ethical’ leadership are rooted in research that assesses the characteristics of ‘good’ leaders [65]. Attitudes, however, guide behavior; leadership theories have added other categories of ‘moral leaders. Prior to the emergence of ethical leadership, the traits or characteristics of a leader were defined as part of ‘transactional’, ‘transformational’, ‘steward’, or ‘authentic’ leadership approaches [66,67,68]. Kanungo [66] thought that leaders have a ‘moral foundation’ and showed differences and similarities of ethical leadership traits by comparing leadership traits to the theories of transformational and transactional. Both perspectives have a common foundation, but differ in characteristics and styles; however, according to Kanungo, both styles act as ethical leaders. While transactional leadership focuses on work, purpose, and ethical behavior, transformational leaders focus on ethics, principles, and social values. Bass and Steidlmeier [65] argued that leaders with transformational orientation, encourage change through vision, and are considered ethical. However, this is partly based on subjective assumptions. The same can be the case for values-based leaders. Introduced by Avolio and Gardner [69], over time this perspective changes from the view of ‘values-based’ to ‘moral’ leaders. This research domain focuses on social norms (business and culture) and human values and the development of cognition and morality [70]. Previous studies have focused on ‘values-based’ approaches, placing emphasis on the problem that organizational context can contradict with thee good intentions [71]. Here, the focus is on ‘near-action’ issues: on the specific actions and choices leaders make in situations and contexts, which can be replaced by ‘intention’ or ‘avoidance’ from the action ’ideas’, such as values and behaviors.

Another research stream, authentic leader, focuses on honesty in relation to the values of true leaders and the actual behaviors of leaders. An authentic leader adheres to cultural norms and precedents, standards, and goals. While integrity has been shown to be the critical priority for ethical leaders, self-awareness and the development of others are also important [72]. However, the very nature of leadership often results in a moral crisis. The true purpose of values-based, ethical, or authentic styles of leadership is often influenced by background and context. In order for it to be validate, good knowledge is needed, as indicated by Brown and Treviño [72]. Behavioral studies cite integrity, commitment, and motivation of others, as guiding principles for a leader’s behavior [73,74]. This concept is also similar to the servant leader and stewardship approach. In their study, Mihelic et al. [73] concluded that an ethical leader is more concerned with avoiding inappropriate leadership. CSR is also intended to prevent misconduct; however, it can be assumed that ethical leadership is the most effective way to solve ethical issues at the workplace and that leaders are the main source of ethical guidance. Importantly, ethical leadership is considered to be one solution for creating a balance between the wellbeing of the employees and the wider community, and the organization’s profitability. Moreover, from the viewpoint of Blake and Mouton’s Managerial Grid [75], team management was found to be the most effective leadership style. This style has similarities with ethical style of leadership; for example, both models (ethical leadership and team management) focus on trust, respect, and empowerment. All these factors eventually create a work atmosphere in which employees feel satisfaction, and thus, are motivated to improve the overall efficiency of their organization.

Despite satisfying different stakeholders, a socially responsible organization also emphasizes building ethical leadership to promote ethicality at the workplace and earn a real meaning of CSR [76]. An organization with a CSR orientation is likely to strengthen its management and leadership with the highest standards of workplace ethics. There is no doubt in accepting the fact that an ethical manager shows a severe concern towards sustainability and environment management [77]. An ethical leader in a socially responsible organization is likely to infuse ethicality among its followers (employees in the current case) as they observe their leader’s actions as a role model [78]. Every enterprise understands the importance of corporate leaders to achieve different organizational objectives effectively [79]. Especially in the current context, a socially responsible organization assumes its management as a critical resource to pursue different business strategies effectively.

Moreover, a socially responsible organization treats its corporate leaders with respect and transparency. All this process boosts this stance of an organization to the management that they belong to a socially responsible organization, and hence, their organization expects its leadership with ethical practices [76]. Thus, promoting ethicality at the workplace is one of the hallmarks of a socially responsible organization [80]. The positive relationship between CSR and ethical leadership has been reported by different researchers recently [77,81,82]. In sum, CSR as a business philosophy can be helpful to deliver some significant manifestations for all stakeholders through sustainable practices and ethical business values. Following ST, the CSR orientation of a socially responsible organization is assumed to benefit society, and the leadership of such an organization becomes self-motivated to exchange this benefit with benefits. Thus, they are expected to act more ethically while performing different tasks at the workplace. Therefore, the following hypothesis is proposed:

**Hypothesis** **2** **(H2).** *There exists a positive relationship between CSR and ethical leadership*.

### 2.4. Ethical Leadership and Pro-Environmental Behavior

As mentioned in the seminal work of Brown and Treviño [72], the normative act of an ethical leader is primarily focused on others’ well-being. In an organizational context, an ethical leader is likely to stress the well-being of their followers instead of stressing personal interest and well-being [83]. Ethical leaders take care of their followers through their ethical conduct at the workplace [84]. Ethical leaders in a socially responsible organization understand that their organization is a caring organization for all stakeholders, including the employees. Therefore, they pay extra attention to the well-being of their employees [85]. Employees are a strategic key resource for any organization to achieve different business objectives. Realizing this importance of employees, an ethical leader is expected to direct their efforts to the interests of the employees [86]. In the context of the current study, the ethical conduct of an ethical leader is well observed by the employees of an organization. As the employees consider their leader a role model, they copy the ethical conduct of the leader. Thus, employees also become more responsible and ethical while performing their job in an organization. Generally, the available literature regards a positive association between ethical leadership and pro-environmental behavior [87,88,89].

Moreover, the CSR orientation of an organization is an extra-role commitment for the society and the environment from a socially responsible organization, which is considered a social benefit by the employees. Thus, following ST, the employees want to exchange this benefit positively. Therefore, they also support their organization beyond its day-to-day activities by performing different discretionary roles, one of which is their pro-environmental behavior. Past literature mentions employees’ CSR perceptions of an organization can be better explained in the presence of an ethical leader [90,91]. One possible reason for this association is that an ethical leader tries to make it certain that their organization’s CSR commitment is communicated at all levels [92]. Owing to this fact, it is mentioned in the mainstream literature that CSR should be contingent on an organization’s culture derived from organizational values, norms, and ethics [93,94,95]. Employing a proper leadership style (ethical leadership in the current case) may shape the environment-specific behavior of employees in a socially responsible organization [96]. Mentioning this fact, Afsar et al. [97] argued that the responsible behavior of a leader further strengthens the CSR perception of employees. Leaders with ethical conduct advocate for the environment and continue mentioning the importance of a sustainable environment for future generations [98]. Ethical leaders are critical strategic enablers to shape the environment-specific behavior of their followers in an enterprise [99]. It is highly likely to expect that the CSR-oriented conduct of an ethical leader is mimicked on the part of employees, and they are self-motivated to show a positive commitment to the environment through their pro-environmental behavior. On a final note, in line with ST, when employees of a socially responsible organization observe the ethical conduct of their leader, they want to exchange this ethicality and the socially responsible behavior of their organization positively. Thus, they are expected to promote environment-friendly behavior on their part too. Moreover, the presence of an ethical leader makes it possible for every employee to realize their importance in reducing the overall environmental footprint of an organization. Thus, the following hypotheses may be proposed:

**Hypothesis** **3** **(H3).** *There exists a positive relationship between ethical leadership and employees’ pro-environmental behavior*.

**Hypothesis** **4** **(H4).** *Ethical leadership mediates between CSR and employees’ pro-environmental behavior*.

### 2.5. Gender as Moderator

Social role theory is grounded in the proposition that individuals are socially classified as male or female and tend to possess different ascribed roles inside a given social structure. Their role is judged against divergent expectations for how they should behave [100]. Gender roles are socially constructed roles covering a range of behavior, actions and attitudes that are desirable, acceptable, and suitable for a person according to their biological sex [101]. Gender roles are generally connected with the notion of masculinity and femininity [102]. In a patriarchal society, gender discrimination and socio-economic inequality make women a marginalized group, lowering their voice and power. Empowering women by ensuring gender equality and reducing socio-economic imbalances may be the best means of protecting the environment [103]. Women generally care more about environmental problems and have staunch pro-climate and environment caring notions and beliefs [104]. Women in public offices are expected to be more inclined to sign agreements and treaties on reducing global warming and climate change than men.

Although women suffer from environmental disasters and climate change, they have the awareness and capability to find indigenous environmental problems solutions. Therefore, such knowledge and skills should be aligned with environmental programs, policies, and finance while supporting females in the presence of unprecedented environmental occurrences.

Some socio-psychological researchers suggest that females’ reproductive and nurturing role leads towards a caring attitude for others and demonstrate behavior pertinent to environmental well-being [19,105]. In continuous environmental degradation, it is crucial to protect the environment and save natural resources. Women tend to be more directly involved in the management and use of natural resources that come from the environment. Women play a vital role in the allocation of resources to family and community and even to organizations. That is why they have direct stakes in the environment and become much influenced by the natural environment. Thus, conservation of the environment and protection of natural resources cannot be realized without women’s participation in decision making and training to frame standards to protect the environment [20].

**Figure 1:**
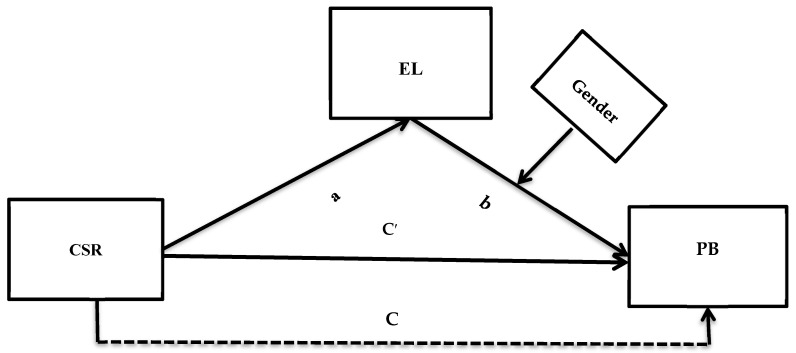
Hypothesized framework of the current study. CSR (X) = the independent variable, pro-environmental behavior―PB (Y) = the dependent variable, ethical leadership―EL (M) = the mediating variable, Gender = moderating variable, C′ = direct effect of X on Y in the presence of mediator, C = direct effect of X on Y without mediator. a and b represent the slope.

Recently, scholars have stressed that CSR engagement of an organization not only improves the environmental footprint of an organization but also induces its economic efficiency. For example, the recent study of Al-Shammari et al. [106] showed that the organizations that fulfill their economic and social responsibilities realize better financial outcomes compared to their rivals. The same was also proved in the work of Kim et al. [107], who employed a competitive-action perspective to propose the positive relationship between CSR and the financial performance of an organization. In the current context, the dual responsibility of the organization makes female leaders probably more interested in promoting themselves as leaders who care about these two types of responsibilities of a business. Moreover, studies on women and the environment have shown that women are very close to nature and are the agents of natural resource management, and they are major sponsors to environmental conservation and rehabilitation [108]. It has been noted that women have competencies in managing natural resources. The role of females as a leader remained a part of academic debate since 1975; however, their numbers in executive positions did not rise significantly in the corporate world in the past [109]. Conventionally, it was established that females were less likely to be associated with leadership roles and that females in leadership positions are more stereotyped compared to male leaders [110,111]. Moreover, it was also mentioned that females are more emotional than males; thus, their leadership role was not encouraged in the past [112]. At the same time, it was also reported that the ‘caring for others’ value is higher in females than men [113,114]. Recently, KSA has witnessed significant changes, and the role of females for different management and leadership positions in public spheres was brought to the fore [115]. Moreover, the current regime led by King Salman has witnessed significant social reforms to empower females in the country. This is why females now capture a better representation for leadership positions than in the past [116]. In the current perspective, females have shown to be good managers of natural resources and more sensitive towards environmental issues, as they possess a high level of ecological consciousness. Moreover, the literature generally establishes that women as corporate leaders are better promoters of the environment than men [117,118]. Thus, keeping in mind these theoretical assumptions and logical linkages, the following hypothesis can be proposed. Please refer to Figure 1 for detail.

**Hypothesis** **5** **(H5).** *The role of gender moderates the mediated relationship between CSR and employees’ pro-environmental behavior such that in the case of women, the relationship is stronger as compared with the case of men*.

## 3. Methodology

### 3.1. Sample and Data Collection

The healthcare system in KSA is primarily a nationalized healthcare system that the government of centrally operates. The ministry of health is the major regulator of the healthcare system in the kingdom. Through its dedicated funding, the government of KSA makes it possible for every citizen to have free access to a healthcare facility throughout the kingdom [119]. Besides the national healthcare structure, recently, there has been growing participation from the private sector in KSA. The constitution of KSA declares healthcare facilities as a basic right [120]. KSA has a clear commitment towards a welfare policy and universal access to healthcare [121].

Equally important to mention here is that, as per the guidelines of WHO, the air quality in KSA is considered unsafe in many cities. Moreover, the recent data mentioned that KSA’s annual mean concentration of PM2.5 is 88 µg/m^3^, far beyond the threshold value of 10 µg/m^3^ [122]. Emissions from vehicles and industries have significantly contributed to this poor air quality index. The cities of Riyadh, Jeddah and Ghran are cities with high levels of air pollution. Therefore, the current study considered the hospitals located in these cities for data collection.

In this regard, the authors first made an official communication with the concerned departments of the targeted hospitals to seek their formal permission to collect the data from their staff in the best interest of academia and industry. These departments included the human resource department or the department dealing with external affairs of a hospital (such as PR departments, among others). The hospitals were selected after verifying their CSR engagement in different social activities. The hospitals that showed initial commitment to facilitate the authors in data collection were then approached by the authors to settle down different arrangements of data collection (scheduling, timing, dates, etc.). After addressing these arrangements, the authors were finally able to maintain their presence in the premises of specified hospitals. It is important to mentioned here that the data were collected from January to March 2021.

Before the data collection process, the authors followed the ethical guidelines of the Helsinki declaration [123]. In this regard, each respondent was served with an “informed consent” sheet to participate in the survey voluntarily. Moreover, each respondent was communicated by the authors that they can quit the survey at any stage without explaining any specific reason to the authors if they feel uncomfortable in disclosing the information. Such steps were taken to ensure the ethical standards during this survey.

The current survey instrument was an adapted questionnaire that was evaluated by the experts in the field for suitability and appropriateness before providing it to the respondents. These guidelines are in line with the recommendations of Guo et al. [124]. The questionnaire items were written in English, although Arabic is the national language. However, given that hospital staff holds a considerable formal education, understanding an English language questionnaire was not an issue. The questionnaires were distributed among the hospital staff randomly. The staff included healthcare professionals (doctors, nurses, and allied services staff) and the administrative staff (general administration of a hospital). More specifically, to identify the gender of a leader in a particular department or unit, the respondents were given to encircle either a letter “W” or “M”.

A total of 700 questionnaires were initially distributed among different respondents, and eventually, 489 valid responses were received back, which were included in the final dataset of the current study. This implies that the response rate of the current study remained 69.85%. For more demographic-related details, see Table 1 below.

### 3.2. Measures

The current study used the existing scales to operationalize different constructs, including CSR, employees’ pro-environmental behavior, and ethical leadership. The authors used these existing scales because of their pre-established reliability and validity [27,125]. In this regard, the authors adapted a 12-item scale of CSR from Turker [126]. This scale is a well-reputed scale for measuring CSR perception. Different extant researchers have also employed this scale in recent studies [97,124]. A sample item of this scale was “This hospital encourages its employees to participate in voluntary activities”. Similarly, the ethical leadership scale was adapted from the study of Brown et al. [127]. There were 10-items to measure ethical leadership. A sample item was “Our leader sets an example of how to do things the right way in terms of ethics”. Various scholars have also used this scale in their studies. For instance, the studies of Abuzaid [128] and Alpkan et al. [129] are some evocative examples. Finally, the items of pro-environmental behavior were adapted from Robertson and Barling [130]. This scale was composed of seven items. A sample item was “He/she is a person who turns lights off when not in use”. A five-point Likert scale was employed to record the responses of the respondents.

## 4. Results

### 4.1. Common Method Bias

Common method bias (CMB) is a common issue in survey methods of data collection [131]. The presence of CMB makes it possible that the variation in the responses collected by a researcher is associated with a biased instrument rather than the actual predisposition of the respondents. Therefore, the issue of CMB may lead a researcher to draw misleading results because such results happen due to a biased instrument and not due to the changes in perception of the respondents [132]. As the current study collected the data through a questionnaire (survey design), the authors decided to confirm the absence of CMB into the dataset before proceeding further in the data analysis process. The common approach to detect CMB is to perform a single factor test as recommended by Harman [133]. To do this, the authors loaded all the items onto a single factor without using any rotation in SPSS software. The results of this analysis are (Table 2) helpful to detect the presence of CMB in a dataset. For this, if the results of the single-factor analysis confirm the presence of a factor that explains a significant proportion of total variance (50% or above), then there exists the issue of CMB. In the current case, no such dominant single factor emerged during the single factor analysis, implying that CMB is not a potential issue in the current case. The largest variance explained by the single factor was 33.79% which is well below the threshold level of 50%. Nevertheless, to further cement the observation that the current survey data do not suffer from the issue of CMB, the authors also performed a single confirmatory factor analysis (CFA) to cross-validate that there is no issue of CMB. Again, the outcomes of such single CFA revealed a poor model fit, implying that the one-factor model is not the appropriate model (*χ*^2^ = 1925.462, *df* = 289, *χ*^2^/*df* = 6.662, RMSEA = 0.089, CFI = 0.53, NFI = 0.47).

### 4.2. Construct Evaluation: Factor Loadings, Validity, and the Reliability

The confirmation for the non-existence of CMB paved the way for authors to continue with the data analysis at a further level. Hence, this time the authors performed different tests (factor loadings, validity, and reliability tests) to evaluate all constructs of the current survey. To do this, in the first place, the authors checked the factor loadings of all items. In this regard, the standard criterion to declare an item’s loading as valid is *λ* > 0.5, and ideally, it should be greater than 0.7. As can be seen from the output of Table 3, all the items’ loadings show good values (as nothing less than 0.5 was observed). Therefore, no issues were signaled regarding the factor loading of any item.

Likewise, in the second place, to establish the validity of each construct, the authors calculated the average-variance-extracted (AVE) for each construct by using the formula given in Equation (1):

(1) $$\mathrm{AVE}=\frac{\sum_{i=1}^{k}\lambda_{i}^{2}}{\sum_{i=1}^{k}\lambda_{i}^{2}+\sum_{i=1}^{k}.var\left(\varepsilon i\right)}$$

At this stage, the authors employed the criterion of Fornell and Larcker [134] for the AVE of each construct. According to them, ideally, the value of AVE for a construct should be 0.5 or beyond to establish that a majority of variance in a construct is explained by its items and not by the error terms. As for the AVE results for all three constructs, i.e., CSR, ethical leadership (EL), and pro-environmental behavior (PB), all were above the cut-off value of 0.5. For instance, it can be seen in Table 3 that the value of AVE for PB is 0.679, implying that the condition for convergent validity is fulfilled. These guidelines to validate a construct can also be seen in the seminal work of Gefen et al. [135].

In the third place, the authors also evaluated the composite reliability (C_rl_) for each construct. To achieve this, the formula in Equation (2) was employed for such calculations:

(2) $$\mathrm{Composite}\;\mathrm{reliability}=\left(\left(\sum \lambda_{i}\right)2\right)/\left(\sum \lambda_{i}\right)2+\sum var\left(\varepsilon i\right))$$

In this regard, a C_rl_ value beyond 0.7 is considered adequate to establish the reliability of a construct. The results of C_rl_ have also been reported in Table 3 for the convenience of the readers.

In the next stage of the data analysis, the authors further performed several statistical tests. For example, the authors performed correlation analysis to assess the nature and value of correlation among the constructs (Table 4). The results of this analysis acknowledged a positive relationship among different variables. This positive association is a good indication towards the validation of different hypotheses of the current study. To explain further, one can see the value of correlation between CSR and EL is *r* = 0.462, which is positive and significant, implying that there exists a positive association between CSR and EL. The same is the case with other pairs of correlations.

Meanwhile, in order to validate the discriminant validity, the authors employed two tests. Firstly, the authors calculated the maximum shared variance (MSV) values and average shared variance (ASV) for each construct. The standard rule here is that if both values (MSV and ASV) are less than the value of AVE for the same construct, then the condition for discriminant validity is fulfilled.

As shown in Table 4, all the values of MSVs and ASVs are less than the values of AVE. For instance, the MSV and ASV for CSR are 0.25 and 0.23, far less than the AVE values (0.658). Secondly, to further validate discriminant validity, the authors also calculated the square root of every construct and compared it with AVE in this respect. If the value of the square root of AVE for a construct is greater than the values of correlations, then discriminant validity is maintained. For instance, the value of the square root of AVE for CSR is 0.811, which is greater than the correlation values (0.462 and 0.498) Hence, it is validated that there is no issue in discriminant validity and the items of one construct are dissimilar from the items of the other construct [136]. Lastly, the authors also verified the model fitness of the measurement model of the current survey. In doing so, some important model fit indices (MFI) values were assessed their standard ranges again. For example, the standard range of *χ*^2^/*df* is less than 3. In the current case, this value was (1719.238/792) 2.171, less than 3. Likewise, the normed fit index (NFI) value was 0.929, which is greater than the standard range of 0.90. Moreover, the comparative fit index (CFI) value should also be greater than 0.90, which is 0.936 in the current case. Likewise, the root means square of error approximation (RMSEA) value should be less than 0.08, which was observed as 0.049. All these results validate that there is a good fit between theoretical model and the measured model (*χ*^2^ = 1719.238, *df* = 792, *p* < 0.05, *χ*^2^/*df* = 2.171, RMSEA = 0.049, CFI = 0.936, NFI = 0.929).

### 4.3. Hypotheses Validation

After performing several statistical tests and construct validation, the authors tested the hypotheses of the current study by performing structural equation modeling (SEM). Specifically, the SEM analysis was employed in several steps. In the first place, a structural model was developed to test the direct association of the hypotheses (H1, H2, and H3). The results of this analysis have been reported in Table 5. According to these results, the first three hypotheses (H1, H2, and H3) were statistically proved to be true (β1 = 0.426, β2 = 0.388, β3 = 0.329, *p* < 0.05); hence, H1, H2, and H3 are accepted. This decision was reached based upon the positive beta values (β1, β2, and β3). Moreover, the p-value in each case was also significant. Similarly, both the upper limit confidence interval (ULCI) and the lower limit confidence interval (LLCI) for all three hypotheses do not include any zero value, establishing that H1, H2, and H3 are accepted (*χ*^2^ = 1529.438, *df* = 828, *p* < 0.05, *χ*^2^/*df* = 1.847, RMSEA = 0.040, CFI = 0.942, NFI = 0.934).

After validating the structural model for direct relationships (H1, H2, and H3), the authors tested the mediation results (H4). For this purpose, the previous structural model was modified, as this time, the EL was included as a mediator in the structural model. Moreover, the bootstrapping option was also employed to confirm the mediation effect of EL. For this, a large bootstrapping sample of 2000 was considered by the authors. Using this large bootstrapping sample is also recommended in several studies [27,137,138].

The results of mediation analysis have been reported in Table 6. As per the results given in Table 6, there is a confirmation of the mediation role of EL between CSR and PB. According to the results, the beta value (β4 = 0.128, *p* < 0.05) was positive and significant, implying that H4 is accepted. Moreover, the results of the mediation effect also supported that there is a partial mediation effect of EL between CSR and PB. Furthermore, EL as a mediator caused almost 30% variations in PB. Thus, these results confirmed that EL mediates between CSR and PB (*χ*^2^ = 1323.844, *df* = 779, *p* < 0.05, *χ*^2^/*df* = 1.699, RMSEA = 0.037, CFI = 0.947, NFI = 0.939).

Lastly, to confirm the moderation effect of gender on the indirect relationship of CSR and PB, the authors selected the multi-group option in AMOS software. In doing so, the data for male leaders and female leaders were split into two parts. This time, in the mediated structural model, the authors checked the moderation effect of both genders (male/female) separately. These results have been given in Table 7, and according to these results, both male and female leaders are producing a positive moderation effect between the indirect relationship of CSR and PB. However, in the case of female leaders, this relationship is stronger compared to male leaders. These results imply that the female leadership is producing a more convincing effect to shape the environment-specific behavior of employees. Thus, H5 of the current study is also accepted.

## 5. Discussion

There were four major objectives to carry out in the current study. Firstly, the study intended to investigate the factors that influence the pro-environmental behavior of employees in the healthcare sector of developing countiries. To this end, the current study’s findings bring to the fore that CSR perception of employees about their organization supported by ethical leadership can spur the discretionary behavior (pro-environmental) of employees. Such findings are also in line with prior studies [97,139]. The survey respondents confirmed that they are self-motivated to participate in different environment-specific activities at the workplace in response to the CSR commitment of their organization and the ethical conduct of their management. Secondly, the study attempts to extend the discussion on CSR from the perspective of environmental management. To this extent, the findings supported that CSR perception of employees working in different socially responsible hospitals can inculcate in employees’ positive feelings about their organization. These results can also be explained in light of the social exchange theory. As stated earlier, the individuals are expected to continue a social relationship with others by exchanging the benefits received from others with benefits. In this regard, when employees observe the CSR orientation of their socially responsible organization for society and the environment, they take such participation of an organization as a benefit for society, the environment, and the employees. In response, they feel the motivation to exchange this benefit (CSR) received by their organization, with a benefit. Thus, they become more pro-social at the workplace and willingly take part in different environment-specific activities to support their organization to preserve nature. Specifically, as CSR engagement of an organization is assumed as a discretionary commitment of an organization for all stakeholders, this discretionary commitment is exchanged by the employees (a key stakeholder) by their engagement into certain discretionary tasks. Among these, their pro-environmental behavior is also discretionary behavior. These findings were also supported in the seminal work of Glavas [140] and Jones et al. [141].

Thirdly, the current study also highlights an ethical leader’s importance in explaining the association between CSR and employees’ pro-environmental behavior. In this regard, as per the findings of the current analysis, it was observed that in the presence of ethical leadership, the followers extend their environmental commitment to a further level. Especially when they observe that their leader is an advocate of ethicality at the workplace, they copy the ethical conduct of the leader as a role model. Hence, these processes help them to shape their ethical behavior at the workplace. Generally, a relationship of CSR and employees’ positive behavior (pro-environmental behavior in the current case) exists; however, in the presence of an ethical leader, this relationship provides an added explanation to the employees to participate in different environment-specific behaviors. More importantly, an ethical leader communicates to the follower about their importance to improve the overall environmental footprint of their organization through the voluntary engagement of the employees. To this end, the study confirms the mediation role of an ethical leader to explain a positive association between CSR and employees’ pro-environmental behavior. Recently, some other scholars have also mentioned the mediating effect of leadership to explain the discretionary behavior of employees [142,143].

Fourthly, another important objective of the current analysis was to discuss the role of gender difference for the indirect relationship of CSR and employees’ pro-environmental behavior. Specifically, the current study intends to highlight the moderating role of a female leader to better support the above-stated indirect relationship. As per the findings of the current analysis, it was observed that both male and female leadership moderate the indirect relationship of CSR and pro-environmental behavior. However, the case of a female leader is well-differentiated from the case of male leaders, as in the presence of a female leader, the indirect relationship of CSR and employees’ pro-environmental behavior is stronger than male leaders. Generally, the prior literature also regards females’ important role in preserving the environment [117,118,144]. Recently, developing country like KSA has witnessed significant changes, and the role of females in different management and leadership positions in public spheres was brought to the fore [115]. Moreover, the current regime led by King Salman has witnessed significant social reforms to empower females in the country. This is why females now capture a better representation for leadership positions than in the past [116], implying that there is a paradigm shift in the culture of KSA to empower females and realize their leadership potential in the corporate world. Additionally, the theory of social role also supports this concern of females for the environment. More specifically, the scholars regard the correspondent inferences that females are more caring individuals [145]. Moreover, individuals carry out specific gender roles as they enact specific social roles. Socialization is a mere facilitator for such gender-oriented role performances [146]. Thus, the role of female leaders is more important for infusing pro-environmental behavior among employees than male leaders.

### 5.1. Implications

#### 5.1.1. Implications for Theory

From a theoretical point of view, the current study enriches the literature on organizational management and CSR in three ways. In the first place, the current study enriches the literature on CSR from the perspective of employees. In this regard, most of the current literature assumes CSR from the perspective of organizational outcomes, including organizational performance [54,82] or organizational reputation [49]. Knowing the potential benefits of CSR at the level of employees is more important, especially from the perspective of environmental management. However, such contributions in the literature are relatively sparse. The current study is an attempt against this backdrop.

In the second place, the current study also stresses the mediating role of ethical leadership to better explain the relationship between CSR and pro-environmental behavior. In this regard, very few studies have attempted to investigate the mediating effect of a leadership style to shape the behavior of employees. To our knowledge, only the studies of Molnár et al. [21] and Murtaza et al. [27] have followed a similar approach. Nonetheless, many studies have highlighted the direct importance of a leadership style, especially an ethical style of leadership, to spur employees’ pro-environmental behavior [81,88]. However, the academic debate has paid relatively little attention to the potential mediating effect of ethical leadership on employees’ discretionary behavior.

Finally, and most importantly, the current study highlights the important role of gender, especially the role of a female leader, to spur the indirect relationship of CSR and employees’ pro-environmental behavior. Although there have been different studies highlighting the importance of females to protect the environment [108,117], few studies have analyzed the concept in the context of a patriarchal culture. Thus, the current study is one of the few that attempts to highlight the important role of females for better management of the environment.

#### 5.1.2. Implications for Practice

The current study has some critical practical implications for the healthcare industry. First of all, the current study highlights the importance of CSR engagement in a hospital from the perspective of environmental management. As the findings of the current study unveiled, the CSR perception of hospital employees plays an important role in shaping their eco-friendly behavior. Realizing this fact, this is the time for the policymakers of this sector to reframe the strategic intent of CSR as the current state of CSR affairs in most hospitals is related to charity and donations that cannot help a hospital improve its environmental footprint. A hospital needs to understand the potential benefit of CSR to address the natural environment. In this regard, the Kingdom of Saudi Arabia can benefit from the example of the nations of the EU, which were able to improve their natural environment by promoting its importance on an individual level. Due to such initiatives of the EU, individuals recognize their important role in improving the environment, which is perhaps why the sustainability index of most EU countries improves each year.

Secondly, the current study also highlights the importance of an ethical leader for a hospital. In this regard, it is important to mention here that only the CSR engagement of an organization is not enough. The presence of responsible management is a necessary precondition to achieve the expected outcomes for a hospital. Moreover, an ethical leader further highlights the importance of the individual to improve nature and the environment. Especially in the context of sustainability management, the presence of an ethical leader makes their role toward environmental improvement clear to every employee. There is a dire need to promote such an ethical leadership style in the healthcare sector to address environmental dilapidation effectively.

Lastly, as stated earlier, females are almost half of the population of the kingdom. However, unfortunately, their role in most social development programs is very passive. Given that the KSA is a society characterized by a patriarchal culture, the participation of females in society-building activities is not encouraged. To this end, the results of the current study may be eye-opening for this society to understand that without effective participation on the part of females, it is less likely for the kingdom to achieve its expected sustainability outcomes. Though the government has recently shown seriousness in reducing its carbon footprint, if half of the population remains passive, achieving such objectives will remain a difficult task. Moreover, in light of the findings of this analysis, it is important to promote females in more managerial posts to shape the behavior of a workforce better. Surprisingly, females’ role in the environment did not receive any significant attention in past studies. Moreover, as mentioned by Byun and Al-Shammari [147], the age of female leaders and the power of the CEO can influence the ethical behavior of a female leader, and this context needs to be further explored.

### 5.2. Limitations and Future Research Directions

Even though the current analysis brings to the fore some important implications for the healthcare sector, the study also encounters some important limitations. First of all, the study sample was only limited to three specific cities of one country. Thus, the geographic concentration may limit the generalizability of the current analysis. In this respect, future researchers should include more cities and provinces to make a better and generalizable impact. Secondly, the study only records the perceptual measures of CSR. Although such measures are considered helpful by a plethora of studies, using an objective measure of such constructs in the upcoming studies may generate more realistic outcomes.

Similarly, the current study’s findings may remain limited in its scope because a cross-sectional survey design was employed, limiting the causality of association among different constructs. In this regard, a better approach in future studies may be to incorporate a longitudinal data design. Moreover, given that CSR is context- and culture-specific, the current survey findings may remain the same for similar cultures (UAE, Oman, Qatar, etc.). However, in different cultures, due care is necessary before interpreting the current survey results. Lastly, it would be interesting for future researchers to explore whether the age of the female leaders (early in their career vs. late in their career) or CEO power (which either enables or hinders the influence of the leader) may have a differential impact on the ethical behavior of the female leader.

## 6. Conclusions

The current study is one of those few studies that considered the importance of CSR to improve the environmental footprint for a nation that is already a victim of climate vulnerability. The study attempts to elucidate for interested policymakers that assuming CSR from the perspective of the environment may be a strategic enabler. More specifically, the study also indicates the importance of female leaders to achieve the sustainability objectives. Unfortunately, despite their presence in the healthcare and education sector, females barely have any significant presence in other fields of activity. In this respect, the findings indicate the critical importance of an appropriate presence in every sector.

## Figures and Tables

**Table 1:** Demographic information of the respondents.

Demographic	Frequency	%
Gender		
Men	278	56.85
Women	211	43.15
Age (Year)		
20–25	69	14.11
26–30	138	28.22
31–40	127	25.97
41–50	98	20.04
Above 50	57	11.66
Experience (Years)		
2–4	76	17.3
5–7	188	39.6
8–10	142	30.7
Higher	83	12.4
Category		
Healthcare professionals	302	61.76
General administration	187	38.24

Note: total respondents = 489.

**Table 2:** Results of single-factor analysis.

Factor	Initial Eigenvalues			Extraction Sums of Squared Loadings		
	Total	% of Variance	Cumulative %	Total	% of Variance	Cumulative %
1	9.799	33.788	33.788	9.221	31.796	31.796
2	3.061	10.554	44.342			
3	2.518	8.682	53.025			
4	1.511	5.211	58.236			
5	1.262	4.351	62.587			
6	1.122	3.868	66.455			
7	0.967	3.335	69.790			
8	0.790	2.725	72.515			
9	0.738	2.546	75.061			
10	0.694	2.394	77.455			
11	0.588	2.027	79.482			
12	0.575	1.982	81.464			
13	0.551	1.900	83.365			
14	0.508	1.753	85.118			
15	0.473	1.630	86.747			
16	0.414	1.428	88.176			
17	0.402	1.386	89.561			
18	0.367	1.266	90.827			
19	0.359	1.239	92.066			
20	0.320	1.103	93.170			
21	0.313	1.081	94.250			
22	0.283	0.975	95.226			
23	0.253	0.873	96.099			
24	0.242	0.835	96.933			
25	0.230	0.793	97.726			
26	0.208	0.716	98.442			
27	0.163	0.562	99.004			
28	0.157	0.540	99.544			
29	0.132	0.456	100.000			

Note: Extraction Method: Principal Axis Factoring.

**Table 3:** Factor loadings, convergent validity, and composite reliability.

Item	*λ*	*λ* ^2^	E-Variance	∑*λ*^2^	Items	AVE	CR
CSR1	0.77	0.59	0.41				
CSR2	0.79	0.62	0.38				
CSR3	0.88	0.77	0.23				
CSR4	0.76	0.58	0.42				
CSR5	0.82	0.67	0.33				
CSR6	0.91	0.83	0.17				
CSR7	0.72	0.52	0.48				
CSR8	0.86	0.74	0.26				
CSR9	0.77	0.59	0.41				
CSR10	0.79	0.62	0.38				
CSR11	0.76	0.58	0.42				
CSR12	0.88	0.77	0.23	7.897	12	0.658	0.958
EL1	0.93	0.86	0.13				
EL2	0.74	0.55	0.45				
EL3	0.83	0.69	0.31				
EL4	0.88	0.77	0.23				
EL5	0.73	0.53	0.47				
EL6	0.77	0.59	0.41				
EL7	0.94	0.88	0.12				
EL8	0.78	0.61	0.39				
EL9	0.78	0.61	0.39				
EL10	0.83	0.69	0.31	6.791	10	0.679	0.955
PB1	0.72	0.52	0.48				
PB2	0.71	0.50	0.50				
PB3	0.92	0.85	0.15				
PB4	0.73	0.53	0.47				
PB5	0.88	0.77	0.23				
PB6	0.82	0.67	0.33				
PB7	0.70	0.49	0.51	4.339	7	0.620	0.919

Notes: *λ* = Item loadings, CR = composite reliability, ∑*λ*^2^ = sum of square of item loadings, E-Variance = error variance.

**Table 4:** Correlation, discriminant validity, and model fit indices.

Construct	CSR	EL	PB
CSR	**0.923**	0.462 **	0.498 **
EL		**0.957**	0.392 **
PB			**0.922**
Mean	4.17	3.96	4.09
SD	0.39	0.57	0.62
MSV	0.25	0.21	0.25
ASV	0.23	0.18	0.20
Sqrt (A.V.E)	0.811	0.824	0.787

Notes: SD = standard deviation, ** = significant values of correlation, bold diagonal = Cronbach alpha, maximum shared variance = MSV and average shared variance = ASV.

**Table 5:** The results for hypotheses (H1, H2, and H3).

Path	Relation	Estimates	SE	CR	*p*-Value	ULCI	LLCI	Decision
CSR → PB	+	(β1) 0.426 **	0.060	7.10	***	0.492	0.381	Accepted
CSR → EL	+	(β2) 0.388 **	0.053	7.33	***	0.369	0.311	Accepted
EL → PB	+	(β2) 0.329 **	0.068	4.84	***	0.279	0.196	Accepted

Notes: ULCI = upper-limit confidence interval, LLCI = lower-limit confidence interval, **, *** = significant values.

**Table 6:** Mediation results for H4.

Path	Relation	Estimates	SE	Z-Score	*p*-Value	ULCI	LLCI	Decision
CSR → EL → PB	*+*	(β4) 0.128 **	0.026	4.92	***	0.249	0.163	Accepted
Total effect		0.426						
Indirect effect		0.128						
Direct effect		0.298						
Proportion of mediation		0.30						

Notes: ULCI = upper-limit confidence interval, LLCI = lower-limit confidence interval, **, *** = significant values, SE = standard error.

**Table 7:** Moderation effect of leadership (male vs. female) for H5.

Path	Relation	Estimates	SE	Z-Score	*p*-Value	ULCI	LLCI	Decision
CSR → EL → PB	+	β5_m_ 0.133 **	0.023	4.78	***	0.198	0.111	Accepted
		β5_w_ 0.141 **	0.023	6.13	***	0.212	0.197	Accepted

Note: β5_m_ *=* regression estimates for the case of men and β5_w_ *=* regression estimates for the case of women. **, *** = significant values, SE = standard error.

## Data Availability

The data will be made available on request.

## References

[1] Ince F. (2018). International Businesses and Environmental Issues. Promoting Global Environmental Sustainability and Cooperation.

[2] Abeer A., Amgad E. Saudi Arabia Greenhouse Gas Emissions Have Increased by 225% since 1990. https://www.climatescorecard.org/2020/12/saudi-arabia-greenhouse-gas-emissions-have-increased-by-225-since-1990/#_ftn1.

[3] Al-Wabel M.I., Sallam A., Ahmad M., Elanazi K., Usman A.R.A. (2020). Extent of Climate Change in Saudi Arabia and Its Impacts on Agriculture: A Case Study from Qassim Region. Environment, Climate, Plant and Vegetation Growth.

[4] Rivera J., Clement V. (2019). Business adaptation to climate change: American ski resorts and warmer temperatures. Bus. Strat. Environ..

[5] Paes L.A.B., Bezerra B.S., Deus R.M., Jugend D., Battistelle R.A.G. (2019). Organic solid waste management in a circular economy perspective–A systematic review and SWOT analysis. J. Clean. Prod..

[6] Dahlmann F., Bullock G. (2020). Nexus thinking in business: Analysing corporate responses to interconnected global sustainability challenges. Environ. Sci. Policy.

[7] Goyal S., Routroy S., Singhal A. (2019). Analyzing environment sustainability enablers using fuzzy DEMATEL for an Indian steel manufacturing company. J. Eng. Des. Technol..

[8] AlZgool M.R.H. (2019). Nexus between green HRM and green management towards fostering green values. Manag. Sci. Lett..

[9] El-Kassar A.-N., Singh S.K. (2019). Green innovation and organizational performance: The influence of big data and the moderating role of management commitment and HR practices. Technol. Forecast. Soc. Chang..

[10] Groening C., Sarkis J., Zhu Q. (2017). Green marketing consumer-level theory review: A compendium of applied theories and further research directions. J. Clean. Prod..

[11] Jerónimo H.M., Henriques P.L., de Lacerda T.C., da Silva F.P., Vieira P.R. (2019). Going green and sustainable: The influence of green HR practices on the organizational rationale for sustainability. J. Bus. Res..

[12] Fernando Y., Jabbour C.J.C., Wah W.-X. (2019). Pursuing green growth in technology firms through the connections between environmental innovation and sustainable business performance: Does service capability matter?. Resour. Conserv. Recycl..

[13] Ahmad N., Mahmood A., Han H., Ariza-Montes A., Vega-Muñoz A., Iqbal Khan G., Ullah Z. (2021). Sustainability as a “new normal” for modern businesses: Are smes of pakistan ready to adopt it?. Sustainability.

[14] Raza A., Farrukh M., Iqbal M.K., Farhan M., Wu Y. (2021). Corporate social responsibility and employees’ voluntary pro-environmental behavior: The role of organizational pride and employee engagement. Corporate Social Responsibility and Environmental Management.

[15] Ahmad N., Ullah Z., Arshad M.Z., Kamran H.W., Scholz M., Han H. (2021). Relationship between corporate social responsibility at the micro-level and environmental performance: The mediating role of employee pro-environmental behavior and the moderating role of gender. Sustain. Prod. Consum..

[16] Ahmad N., Naveed R.T., Scholz M., Irfan M., Usman M., Ahmad I. (2021). CSR Communication through Social Media: A Litmus Test for Banking Consumers’ Loyalty. Sustainability.

[17] Wang Y., Sun X., Wang B., Liu X. (2020). Energy saving, GHG abatement and industrial growth in OECD countries: A green productivity approach. Energy.

[18] Gaganis C., Pasiouras F., Voulgari F. (2018). Culture, business environment and SMEs’ profitability: Evidence from European Countries. Econ. Model..

[19] Rodda A. (1991). Women and the Environment.

[20] Agbogidi O.M., Ofuoku A.U. (2007). Promoting environmental protection in Nigeria through environmental education: The role of women. J. Environ. Ext..

[21] Molnár E., Mahmood A., Ahmad N., Ikram A., Murtaza S. (2021). The Interplay between Corporate Social Responsibility at Employee Level, Ethical Leadership, Quality of Work Life and Employee Pro-Environmental Behavior: The Case of Healthcare Organizations. Int. J. Environ. Res. Public Health.

[22] Yıldırım M., Dinçer M.A.M. (2016). How the Process of the CSR Activities Works on Private Hospitals: Case Study from Strategic Perspective. Procedia-Soc. Behav. Sci..

[23] Yıldırım M., Dinçer M.A.M. (2020). How the Process of the CSR Activities Works on Private Hospitals and Pharmaceutical Firms: Multiple Case Study from Strategic Perspective. J. Relig. Health.

[24] Ahmad N., Ullah Z., Mahmood A., Ariza-Montes A., Vega-Muñoz A., Han H., Scholz M. (2021). Corporate social responsibility at the micro-level as a “new organizational value” for sustainability: Are females more aligned towards it?. Int. J. Environ. Res. Public Health.

[25] Gerpott F.H., Van Quaquebeke N., Schlamp S., Voelpel S.C. (2017). An Identity Perspective on Ethical Leadership to Explain Organizational Citizenship Behavior: The Interplay of Follower Moral Identity and Leader Group Prototypicality. J. Bus. Ethics.

[26] Dust S.B., Resick C.J., Margolis J.A., Mawritz M.B., Greenbaum R.L. (2018). Ethical leadership and employee success: Examining the roles of psychological empowerment and emotional exhaustion. Leadersh. Q..

[27] Murtaza S., Mahmood A., Saleem S., Ahmad N., Sharif M., Molnár E. (2021). Proposing Stewardship Theory as an Alternate to Explain the Relationship between CSR and Employees’ Pro-Environmental Behavior. Sustainability.

[28] Eagly A.H., Wood W., Diekman A.B. (2000). Social role theory of sex differences and similarities: A current appraisal. Dev. Soc. Psychol. Gend..

[29] Dovidio J.F., Piliavin J.A., Schroeder D.A., Penner L.A. (2017). The Social Psychology of Prosocial Behavior.

[30] Rand D., Brescoll V.L., Everett J.A.C., Capraro V., Barcelo H. (2016). Social heuristics and social roles: Intuition favors altruism for women but not for men. J. Exp. Psychol. Gen..

[31] Alharbi N., Alhaji J., Qattan M. (2021). Toward Sustainable Environmental Management of Healthcare Waste: A Holistic Perspective. Sustainability.

[32] The World Bank Population, Female (% of Total Population)-Saudi Arabia. https://data.worldbank.org/indicator/SP.POP.TOTL.FE.ZS?locations=SA.

[33] Deena K. How Saudi Arabia’s Women Are Pushing into the Workforce and Transforming the Economy. https://www.thenationalnews.com/business/economy/how-saudi-arabia-s-women-are-pushing-into-the-workforce-and-transforming-the-economy-1.1236956.

[34] Khan S.A., Al-Maimani K.A., Al-Yafi W.A. (2013). Exploring corporate social responsibility in Saudi Arabia: The challenges ahead. J. Leadersh. Account. Ethics.

[35] Waddock S.A., Bodwell C., Graves S.B. (2002). Responsibility: The new business imperative. Acad. Manag. Exec..

[36] Blau P.M. (1964). Exchange and Power in Social Life.

[37] Yin N. (2018). The influencing outcomes of job engagement: An interpretation from the social exchange theory. Int. J. Prod. Perform. Manag..

[38] Ahmad N., Scholz M., Ullah Z., Arshad M., Sabir R., Khan W. (2021). The Nexus of CSR and Co-Creation: A Roadmap towards Consumer Loyalty. Sustainability.

[39] Zhang D., Mahmood A., Ariza-Montes A., Vega-Muñoz A., Ahmad N., Han H., Sial M.S. (2021). Exploring the impact of corporate social responsibility communication through social media on banking customer e-wom and loyalty in times of crisis. Int. J. Environ. Res. Public Health.

[40] Ott H.K., Xiao A. (2017). Examining the role of culture in shaping public expectations of CSR communication in the United States and China. Asian J. Public Relat..

[41] Waheed A., Zhang Q., Zafar A.U., Zameer H., Ashfaq M., Nusrat A. (2021). Impact of internal and external CSR on organizational performance with moderating role of culture: Empirical evidence from Chinese banking sector. Int. J. Bank Mark..

[42] Nurunnabi M., Alfakhri Y., Alfakhri D.H. (2020). CSR in Saudi Arabia and Carroll’s Pyramid: What is ‘known’and ‘unknown’?. J. Mark. Commun..

[43] Tilt C.A. (2016). Corporate social responsibility research: The importance of context. Int. J. Corp. Soc. Responsib..

[44] Saeidi A.E. (2019). The Study of Endogenous Corporate Social Responsibility in Saudi Arabia.

[45] Murphy M.J., MacDonald J.B., Antoine G.E., Smolarski J.M. (2019). Exploring Muslim attitudes towards corporate social responsibility: Are Saudi business students different?. J. Bus. Ethics.

[46] Koleva P. (2021). Towards the Development of an Empirical Model for Islamic Corporate Social Responsibility: Evidence from the Middle East. J. Bus. Ethic.

[47] Shabbir M.S., Wisdom O. (2020). The relationship between corporate social responsibility, environmental investments and financial performance: Evidence from manufacturing companies. Environ. Sci. Pollut. Res..

[48] Hoque N., Rahman A.R.A., Molla R.I., Noman A.H.M., Bhuiyan M.Z.H. (2018). Is corporate social responsibility pursuing pristine business goals for sustainable development?. Corp. Soc. Responsib. Environ. Manag..

[49] Kowalczyk R., Kucharska W. (2019). Corporate social responsibility practices incomes and outcomes: Stakeholders’ pressure, culture, employee commitment, corporate reputation, and brand performance. A Polish–German cross-country study. Corp. Soc. Responsib. Environ. Manag..

[50] Nazari J.A., Hrazdil K., Mahmoudian F. (2017). Assessing social and environmental performance through narrative complexity in CSR reports. J. Contemp. Account. Econ..

[51] Gligor-Cimpoieru D.C., Munteanu V.P., Nițu-Antonie R.D., Schneider A., Preda G. (2017). Perceptions of Future Employees toward CSR Environmental Practices in Tourism. Sustainability.

[52] Shahzad M., Qu Y., Javed S.A., Zafar A.U., Rehman S.U. (2019). Relation of environment sustainability to CSR and green innovation: A case of Pakistani manufacturing industry. J. Clean. Prod..

[53] Carroll A.B. (1979). A three-dimensional conceptual model of corporate performance. Acad. Manag. Rev..

[54] Al-Malkawi H.-A.N., Javaid S. (2018). Corporate social responsibility and financial performance in Saudi Arabia. Manag. Financ..

[55] Saeed T., AlAli M.S. (2020). The Effect of Corporate Social Responsibility (CSR) on Banks’ Financial Performance: A Case Study on Saudi Banks. Int. Res. J. Financ. Econ..

[56] Al-Gamrh B., Al-dhamari R. (2016). Firm characteristics and corporate social responsibility disclosure in Saudi Arabia. Int. Bus. Manag..

[57] Al-Ghazali B.M., Sohail M.S. (2021). The Impact of Employees’ Perceptions of CSR on Career Satisfaction: Evidence from Saudi Arabia. Sustainability.

[58] Kraus S., Rehman S.U., García F.J.S. (2020). Corporate social responsibility and environmental performance: The mediating role of environmental strategy and green innovation. Technol. Forecast. Soc. Chang..

[59] Lu J., Wang J. (2020). Corporate governance, law, culture, environmental performance and CSR disclosure: A global perspective. J. Int. Financ. Mark. Inst. Money.

[60] Afsar B., Umrani W.A. (2020). Corporate social responsibility and pro-environmental behavior at workplace: The role of moral reflectiveness, coworker advocacy, and environmental commitment. Corp. Soc. Responsib. Environ. Manag..

[61] Islam T., Ali G., Asad H. (2019). Environmental CSR and pro-environmental behaviors to reduce environmental dilapidation. Manag. Res. Rev..

[62] Nichols A., Richardson J., Pahl S., Jenkin R., Wallace G., Bennallick M. Sustainable practise and behaviour change in healthcare waste management: A review of the literature. Proceedings of the 1st World Sustainability Forum.

[63] Doh J.P., Stumpf S.A. (2005). Handbook on Responsible Leadership and Governance in Global Business.

[64] Poff D.C. (2010). Ethical Leadership and Global Citizenship: Considerations for a Just and Sustainable Future. J. Bus. Ethics.

[65] Bass B.M., Steidlmeier P. (1999). Ethics, character, and authentic transformational leadership behavior. Leadersh. Q..

[66] Kanungo R.N. (2001). Ethical values of transactional and transformational leaders. Can. J. Adm. Sci. Rev. Can. Sci. Adm..

[67] Heres L., Lasthuizen K. (2012). What’s the difference? Ethical leadership in public, hybrid and private sector organizations. J. Chang. Manag..

[68] Treviño L.K., Hartman L.P., Brown M. (2000). Moral Person and Moral Manager: How Executives Develop a Reputation for Ethical Leadership. Calif. Manag. Rev..

[69] Avolio B.J., Gardner W.L. (2005). Authentic leadership development: Getting to the root of positive forms of leadership. Leadersh. Q..

[70] Brown M.E., Treviño L.K. (2003). Is values-based leadership ethical leadership. Emerg. Perspect. Values Org..

[71] Szabo E., Reber G., Weibler J., Brodbeck F.C., Wunderer R. (2001). Values and behavior orientation in leadership studies: Reflections based on findings in three German-speaking countries. Leadersh. Q..

[72] Brown M.E., Treviño L.K. (2006). Ethical leadership: A review and future directions. Leadersh. Q..

[73] Mihelic K.K., Lipicnik B., Tekavcic M. (2010). Ethical leadership. Int. J. Manag. Inform. Syst..

[74] Martin G.S., Resick C.J., Keating M.A., Dickson M.W. (2009). Ethical leadership across cultures: A comparative analysis of German and US perspectives. Bus. Ethics A Eur. Rev..

[75] Blake R.R., Mouton J.S., Bidwell A.C. (1962). Managerial grid. Adv. Manag. Off. Exec..

[76] Zhu Y., Sun L.-Y., Leung A.S.M. (2013). Corporate social responsibility, firm reputation, and firm performance: The role of ethical leadership. Asia Pac. J. Manag..

[77] Lin C.-P., Liu M.-L. (2017). Examining the effects of corporate social responsibility and ethical leadership on turnover intention. Pers. Rev..

[78] Enwereuzor I.K., Adeyemi B.A., Onyishi I. (2020). Trust in leader as a pathway between ethical leadership and safety compliance. Leadersh. Health Serv..

[79] Saleem F., Zhang Y.Z., Gopinath C., Adeel A. (2020). Impact of Servant Leadership on Performance: The Mediating Role of Affective and Cognitive Trust. SAGE Open.

[80] Glavas A. (2016). Corporate social responsibility and employee engagement: Enabling employees to employ more of their whole selves at work. Front. Psychol..

[81] De Roeck K., Farooq O. (2017). Corporate Social Responsibility and Ethical Leadership: Investigating Their Interactive Effect on Employees’ Socially Responsible Behaviors. J. Bus. Ethics.

[82] Saha R., Cerchione R.S., Singh R., Dahiya R. (2019). Effect of ethical leadership and corporate social responsibility on firm performance: A systematic review. Corp. Soc. Responsib. Environ. Manag..

[83] Kalshoven K., Boon C.T. (2012). Ethical leadership, employee well-being, and helping. J. Pers. Psychol..

[84] Bhatti M.H., Akram U., Bhatti M.H., Rasool H., Su X. (2020). Unraveling the Effects of Ethical Leadership on Knowledge Sharing: The Mediating Roles of Subjective Well-Being and Social Media in the Hotel Industry. Sustainability.

[85] Macassa G., McGrath C., Tomaselli G., Buttigieg S.C. (2020). Corporate social responsibility and internal stakeholders’ health and well-being in Europe: A systematic descriptive review. Health Promot. Int..

[86] Chughtai A.A., Byrne M., Flood B. (2014). Linking Ethical Leadership to Employee Well-Being: The Role of Trust in Supervisor. J. Bus. Ethics.

[87] Suganthi L. (2019). Examining the relationship between corporate social responsibility, performance, employees’ pro-environmental behavior at work with green practices as mediator. J. Clean. Prod..

[88] Khan M.A.S., Jianguo D., Ali M., Saleem S., Usman M. (2019). Interrelations between Ethical Leadership, Green Psychological Climate, and Organizational Environmental Citizenship Behavior: A Moderated Mediation Model. Front. Psychol..

[89] Saleem M., Qadeer F., Mahmood F., Ariza-Montes A., Han H. (2020). Ethical Leadership and Employee Green Behavior: A Multilevel Moderated Mediation Analysis. Sustainability.

[90] Choi S.B., Ullah S.M.E., Kwak W.J. (2015). Ethical Leadership and Followers’ Attitudes toward Corporate Social Responsibility: The Role of Perceived Ethical Work Climate. Soc. Behav. Pers. Int. J..

[91] Hansen S.D., Dunford B.B., Alge B.J., Jackson C.L. (2015). Corporate Social Responsibility, Ethical Leadership, and Trust Propensity: A Multi-Experience Model of Perceived Ethical Climate. J. Bus. Ethics.

[92] Pasricha P., Singh B., Verma P. (2017). Ethical Leadership, Organic Organizational Cultures and Corporate Social Responsibility: An Empirical Study in Social Enterprises. J. Bus. Ethics.

[93] Zhang Q., Oo B.L., Lim B.T.H. (2019). Drivers, motivations, and barriers to the implementation of corporate social responsibility practices by construction enterprises: A review. J. Clean. Prod..

[94] Vveinhardt J., Andriukaitiene R. (2017). Management Culture as Part of Organizational Culture in the Context of Corporate Social Responsibility Implementation. Econ. Sociol..

[95] Upadhaya B., Munir R., Blount Y., Su S.X. (2018). Does organizational culture mediate the CSR—strategy relationship? Evidence from a developing country, Nepal. J. Bus. Res..

[96] Wood B.P., Eid R., Agag G. (2021). A multilevel investigation of the link between ethical leadership behaviour and employees green behaviour in the hospitality industry. Int. J. Hosp. Manag..

[97] Afsar B., Cheema S., Javed F. (2018). Activating employee’s pro-environmental behaviors: The role of CSR, organizational identification, and environmentally specific servant leadership. Corp. Soc. Responsib. Environ. Manag..

[98] Emily N., Akujah P., Okanga P. (2019). Ethical Leadership for Sustainable Development in Developing Countries. Editon Consortium. J. Arts Humanit. Soc. Stud..

[99] Islam T., Khan M.M., Ahmed I., Mahmood K. (2020). Promoting in-role and extra-role green behavior through ethical leadership: Mediating role of green HRM and moderating role of individual green values. Int. J. Manpow..

[100] Kacmar K.M., Bachrach D.G., Harris K.J., Zivnuska S. (2011). Fostering good citizenship through ethical leadership: Exploring the moderating role of gender and organizational politics. J. Appl. Psychol..

[101] Franke G.R., Crown D.F., Spake D.F. (1997). Gender differences in ethical perceptions of business practices: A social role theory perspective. J. Appl. Psychol..

[102] Cramer K.M., Million E., Perreault L.A. (2002). Perceptions of Musicians: Gender Stereotypes and Social Role Theory. Psychol. Music..

[103] Daily B.F., Huang S. (2001). Achieving sustainability through attention to human resource factors in environmental management. Int. J. Oper. Prod. Manag..

[104] Vicente-Molina M., Fernández-Sainz A., Izagirre-Olaizola J. (2018). Does gender make a difference in pro-environmental behavior? The case of the Basque Country University students. J. Clean. Prod..

[105] Dankelman I., Davidson J. (2013). Women and the Environment in the Third World: Alliance for the Future.

[106] Al-Shammari M.A., Banerjee S.N., Rasheed A.A. (2021). Corporate social responsibility and firm performance: A theory of dual responsibility. Manag. Decis..

[107] Kim K.-H., Kim M., Qian C. (2015). Effects of Corporate Social Responsibility on Corporate Financial Performance: A Competitive-Action Perspective. J. Manag..

[108] Lahijanian A., Vaskoei N. (2016). Investigation of empowerment of rural women in environmental protection. J. Environ. Sci. Technol..

[109] Elsesser K.M., Lever J. (2011). Does gender bias against female leaders persist? Quantitative and qualitative data from a large-scale survey. Hum. Relat..

[110] Cecil E.A., Paul R.J., Olins R.A. (1973). Perceived Importance of Selected Variables Used to Evaluate Male and Female Job Applicants. Pers. Psychol..

[111] Terborg J.R., Peters L.H., Ilgen D.R., Smith F. (1977). Organizational and personal correlates of attitudes toward women as managers. Acad. Manag. J..

[112] Shields S.A., Shields S.A. (2002). Speaking from the Heart: Gender and the Social Meaning of Emotion.

[113] Baéz S., Flichtentrei D., Prats M., Mastandueno R., García A.M., Cetkovich M., Ibáñez A. (2017). Men, women…who cares? A population-based study on sex differences and gender roles in empathy and moral cognition. PLoS ONE.

[114] Christov-Moore L., Simpson E.A., Coudé G., Grigaityte K., Iacoboni M., Ferrari P.F. (2014). Empathy: Gender effects in brain and behavior. Neurosci. Biobehav. Rev..

[115] Thompson M.C. (2015). Saudi Women Leaders: Challenges and Opportunities. J. Arab. Stud..

[116] Alotaibi F.T. (2020). Saudi women and leadership: Empowering women as leaders in higher education institutions. Open J. Leadersh..

[117] Glass C., Cook A., Ingersoll A.R. (2015). Do Women Leaders Promote Sustainability? Analyzing the Effect of Corporate Governance Composition on Environmental Performance. Bus. Strat. Environ..

[118] Galbreath J. (2017). Drivers of Green Innovations: The Impact of Export Intensity, Women Leaders, and Absorptive Capacity. J. Bus. Ethics.

[119] GOv.SA Health Care. https://www.my.gov.sa/wps/portal/snp/aboutksa/HealthCareInKSA.

[120] Mufti M.H. (2000). Healthcare Development Strategies in the Kingdom of Saudi Arabia.

[121] Rahman R. (2020). The Privatization of Health Care System in Saudi Arabia. Health Serv. Insights.

[122] IAMAT Saudi Arabia General Health Risks. https://www.iamat.org/country/saudi-arabia/risk/air-pollution#.

[123] PP R. (1964). Human experimentation. Code of ethics of the world medical association. Declaration of Helsinki. Br. Med. J..

[124] Guo M., Ahmad N., Adnan M., Scholz M., Rehman K.U., Naveed R.T. (2021). The Relationship of CSR and Employee Creativity in the Hotel Sector: The Mediating Role of Job Autonomy. Sustainability.

[125] Hyman L., Lamb J., Bulmer M. The use of pre-existing survey questions: Implications for data quality. Proceedings of the European Conference on Quality in Survey Statistics.

[126] Turker D. (2008). Measuring Corporate Social Responsibility: A Scale Development Study. J. Bus. Ethics.

[127] Brown M.E., Treviño L.K., Harrison D. (2005). Ethical leadership: A social learning perspective for construct development and testing. Organ. Behav. Hum. Decis. Process..

[128] Abuzaid A.N. (2018). The relationship between ethical leadership and organizational commitment in banking sector of Jordan. J. Econ. Adm. Sci..

[129] Alpkan L., Karabay M., Şener I., Elçi M., Yıldız B. (2020). The mediating role of trust in leader in the relations of ethical leadership and distributive justice on internal whistleblowing: A study on Turkish banking sector. Kybernetes.

[130] Robertson J.L., Barling J. (2012). Greening organizations through leaders’ influence on employees’ pro-environmental behaviors. J. Organ. Behav..

[131] Podsakoff P.M., Organ D.W. (1986). Self-Reports in Organizational Research: Problems and Prospects. J. Manag..

[132] Chang S.-J., Van Witteloostuijn A., Eden L. (2010). From the Editors: Common method variance in international business research. J. Int. Bus. Stud..

[133] Harman H.H. (1976). Modern Factor Analysis.

[134] Fornell C., Larcker D.F. (1981). Evaluating structural equation models with unobservable variables and measurement error. J. mark. Res..

[135] Gefen D., Straub D., Boudreau M.-C. (2000). Structural equation modeling and regression: Guidelines for research practice. Commun. Assoc. Inform. Syst..

[136] Hair J., Anderson R., Babin B., Black W. (2010). Multivariate Data Analysis: A Global Perspective.

[137] Tse H.H.M., To M.L., Chiu C.K.W. (2017). When and why does transformational leadership influence employee creativity? The roles of personal control and creative personality. Hum. Resour. Manag..

[138] Ogata K. (2021). On the Application of Bootstrapping and Monte Carlo Simulations to Clinical Studies: Psychometric Intelligence Research and Juvenile Delinquency. Psychology.

[139] Tian Q., Robertson J.L. (2019). How and when does perceived CSR affect employees’ engagement in voluntary pro-environmental behavior?. J. Bus. Ethics.

[140] Glavas A. (2016). Corporate social responsibility and organizational psychology: An integrative review. Front. Psychol..

[141] Jones D.A., Willness C.R., Glavas A. (2017). When Corporate Social Responsibility (CSR) Meets Organizational Psychology: New Frontiers in Micro-CSR Research, and Fulfilling a Quid Pro Quo through Multilevel Insights. Front. Psychol..

[142] Wu Q., Cherian J., Samad S., Comite U., Hu H., Gunnlaugsson S.B., Oláh J., Sial M.S. (2021). The Role of CSR and Ethical Leadership to Shape Employees’ Pro-Environmental Behavior in the Era of Industry 4.0. A Case of the Banking Sector. Sustainability.

[143] Shah M.S., Wu C., Ullah Z. (2021). The Inter-Relationship between CSR, Inclusive Leadership and Employee Creativity: A Case of the Banking Sector. Sustainability.

[144] DiRienzo C.E., Das J. (2019). Women in government, environment, and corruption. Environ. Dev..

[145] Eagly A.H., Wood W. (2016). Social Role Theory of Sex Differences. the Wiley Blackwell Encyclopedia of Gender and Sexuality Studies.

[146] Eagly A.H., Wood W. (2011). Social Role Theory. Handbook of Theories in Social Psychology.

[147] Byun K.-A.K., Al-Shammari M. (2021). When narcissistic CEOs meet power: Effects of CEO narcissism and power on the likelihood of product recalls in consumer-packaged goods. J. Bus. Res..

